# Clinical Correlation Between Tumor Maximal Standardized Uptake Value in Metabolic Imaging and Metastatic Tumor Characteristics in Advanced Non-small Cell Lung Cancer

**DOI:** 10.1097/MD.0000000000001304

**Published:** 2015-08-14

**Authors:** Dong Soo Lee, Seung Joon Kim, Hong Seok Jang, Ie Ryung Yoo, Kyung Ran Park, Sae Jung Na, Kyo Young Lee, Sook Hee Hong, Jin Hyoung Kang, Young Kyoon Kim, Yeon Sil Kim

**Affiliations:** From the Department of Radiation Oncology (DSL, HSJ, YSK), College of Medicine, The Catholic University of Korea; Division of Pulmonology (SJK, YKK), Department of Internal Medicine, College of Medicine, The Catholic University of Korea; Department of Radiation Oncology (KRP), School of Medicine, Ewha Womans University; Department of Nuclear Medicine (IRY, SJN), College of Medicine, The Catholic University of Korea; Department of Hospital Pathology (KYL), College of Medicine, The Catholic University of Korea; and Department of Medical Oncology (SHH, JHK), College of Medicine, The Catholic University of Korea, Seoul, South Korea.

## Abstract

This study aimed to elucidate whether the maximal standardized uptake value (SUVmax) of primary tumors in metabolic imaging correlated with pathological or metastatic characteristics and whether it was prognostic in stage IV nonsmall cell lung cancer (NSCLC).

We retrospectively reviewed the medical records of 412 eligible patients between June 2007 and January 2013. All enrolled patients fulfilled the following criteria: they were newly diagnosed with stage IV NSCLC without any previous treatment and had undergone a systemic evaluation, including 18(F)-Fluoro-2-deoxyglucose positron emission tomography/computed tomography, to assess synchronous metastatic sites. Patient and tumor characteristics were analyzed, and clinical correlations between SUVmax and metastatic features were investigated.

The median age of the study population was 65 years (range, 30–94), and 259 (62.9%) patients were male. The median SUVmax was statistically higher in males, in tumors with squamous cell histology, and in poorly differentiated tumors. Multivariate logistic regression analysis revealed that SUVmax ≥ 11.4 (top 30 percentiles) were significantly correlated with positive lymph node status (odds ratio [OR] 3.473), abdomen/pelvis metastasis (OR 1.949), and the absence of bone metastasis (OR 0.399) in the subgroup of nonsquamous NSCLC (n = 343). In Kaplan–Meier survival analysis, overall survival was significantly lower among cohorts with high SUVmax (≥11.4) than with low SUVmax (<11.4) (*P* < 0.001, median 7.4 months vs 12.1 months).

The tumors with different SUVmax have distinctive metastatic and biological features in stage IV NSCLC. The underlying mechanisms of this unique metabolic biology need to be resolved in future studies.

## INTRODUCTION

18(F)-Fluoro-2-deoxyglucose positron emission tomography (18[F]-FDG-PET) has been widely used to make therapeutic decisions, predict prognosis, and accurately diagnose a number of malignancies in humans.^1–6^ The clinical usefulness of 18(F)-FDG-PET in nonsmall cell lung cancer (NSCLC) has been vigorously investigated from multiple points of view.^1–3,7,8^ Yet, these studies have not agreed with one another. Many previous studies explored the prognostic significance of maximal standardized uptake value (SUVmax) in diverse stages of lung cancer^1,7,9,10^: reporting an important association between high SUVmax and poor prognosis,^3,7–10^ but a more recent study failed to demonstrate the similar relationship.^11^ In an updated study of the meta-analysis of 21 studies,^12^ high SUVmax showed a poor prognostic value compared with low SUVmax with an overall combined hazard ratio (HR) of 2.08. However, different studies tested with different stages and treatment factors, and their values in stage IV NSCLC remain uncertain. The present study was performed to examine whether the SUVmax in primary tumors is correlated with pathological or metastatic characteristics and whether it has a prognostic value in a retrospective review of 412 patients with stage IV NSCLC.

## MATERIALS AND METHODS

### Patient Population and Study Methods

We retrospectively analyzed advanced NSCLC patients who had been enrolled in our lung cancer database registry between June 2007 and January 2013. We obtained approval from the Institutional Review Board of the Catholic Medical Center Ethics Committee for this retrospective study. All the enrolled patients were newly diagnosed with stage IV NSCLC^13^ and had no previous treatment for metastatic disease. The exclusion criteria for the study were as follows: patients who did not undergo a systemic work-up, including 18(F)-FDG-PET/computed tomography (CT); patients who were not pathologically confirmed with NSCLC; and patients with no evidence of metastatic disease. Based on these criteria, 412 patients were eligible for inclusion. All eligible patients underwent systemic imaging, including 18(F)-FDG-PET/CT, CT of the whole chest and abdomen, and magnetic resonance imaging (MRI) or CT of the brain to evaluate synchronous metastatic sites. MRI of the spine or Tc-99 m whole-body bone scans were selectively performed to accurately localize skeletal metastatic sites or for other clinical indications. In terms of central nervous system imaging, MRI of the brain was preferentially conducted to detect brain metastases. The smoking status (never smoker vs ex-smoker or current smoker) was routinely evaluated at the first medical examination and was recorded in electronic medical charts.

### Review of Metabolic Imaging and Measurement of SUVmax

The clinical findings of metastatic sites were reviewed by specialized nuclear medicine physicians and radiologists. Before 18(F)-FDG-PET/CT, all patients fasted for at least 6 hours, and a dose of 370 to 555 MBq of FDG was injected intravenously. The CT data used for attenuation correction and PET images were reconstructed with the standard ordered-subset expectation maximization algorithm. The axial spatial resolution was 4.5 mm in the center of the field of view.^14^ The detailed acquisition process for the 18(F)-FDG-PET/CT images was previously described.^3^ The calculation of SUV was carried out using the standard correction for body weight and the injected dose of FDG (SUV = radiotracer concentration/[injected dose/body mass]). The SUVmax was defined as the peak SUV of 1 pixel with the highest count within the tumor in chest and was measured by placing regions of interest around tumors on transaxial views. Accurate discrimination of metastatic disease and reactive inflammatory changes was sometimes challenging. In this situation, symmetrical patterns, the intensity of hot uptake areas, and the serial imaging results or the SUV changes in metabolic imaging were used for exact identification. Based on the metabolic imaging findings, we categorized clinical lymph node (LN) status and metastatic extent for this study. To identify clinical characteristics that were correlated with high SUVmax, we compared variables between the groups in the top rank 30 percentile of SUVmax and the low rank 70 percentile of SUVmax.

### Examination of Tumor Characteristics

We reviewed the histological type of tumor pathology and the differentiation of histologic grading. All patients underwent immunohistochemical staining to discriminate the histological subtypes. The detailed histological subtype was categorized as adenocarcinoma, squamous cell carcinoma, or large cell carcinoma. The remaining tumors with no complete information on specific histology other than NSCLC were categorized as other types.

The categorization of the extent of metastatic tumor was determined for each metastatic site. The synchronous metastatic sites were scored based on the whole-body metastatic score as previously described.^15^ In brief, metastatic sites were classified into 7 groups as follows: abdomen/pelvis (including liver, adrenal gland, LNs, and other abdominal or pelvic organs); lung to lung or systemic lymphangitic spread; bone (skeletal system); pleural and/or pericardial effusion; neck and/or axillary LNs; other soft tissue; and brain. Total scores were calculated from 1 to 7 by summation of each involved region. We further classified metastatic LN status as cN0, cN1, cN2, and cN3 based on the 18(F)-FDG-PET/CT and chest CT. This method was designed to assess whether the SUVmax correlated with metastatic LN status.

### Survival Assessment

Because of the retrospective nature of the study, several patients were lost to follow up from our institution. Thus, the information on overall survival (OS) and the date of death were retrieved from the database registry of our national institution (National Cancer Center, Goyang, Korea). We could extract the final status of all enrolled patients using this method.

### Statistical Analyses

Statistical analyses were conducted using the SPSS statistics (version 12; SPSS Inc, Chicago, IL). For categorical variables (comparison between low and high SUVmax groups), differences among groups were assessed using the Pearson χ^2^ test. There was a nonnormal distribution of SUVmax. Thus, nonparametric tests, such as the Wilcoxon rank-sum test and Kruskal–Wallis test, were used for continuous variables (comparison of median SUVmax). To identify the significant factors associated with the high SUVmax group, univariate and multivariate logistic regression analyses were conducted. The Kaplan–Meier method was carried out to compare survival outcomes using log-rank test. All statistical analyses were based on 2-sided tests and considered statistically significant if *P* < 0.05.

## RESULTS

### Patient and Tumor Characteristics

The patient and pathological tumor characteristics of the 412 patients are shown in Table 1. The median age of the study population was 65 years (range, 30–94), and 62.9% were male. The proportion of never smokers was 39.8%. The majority (73.3%) of patients had an adenocarcinoma histology.

**TABLE 1:**
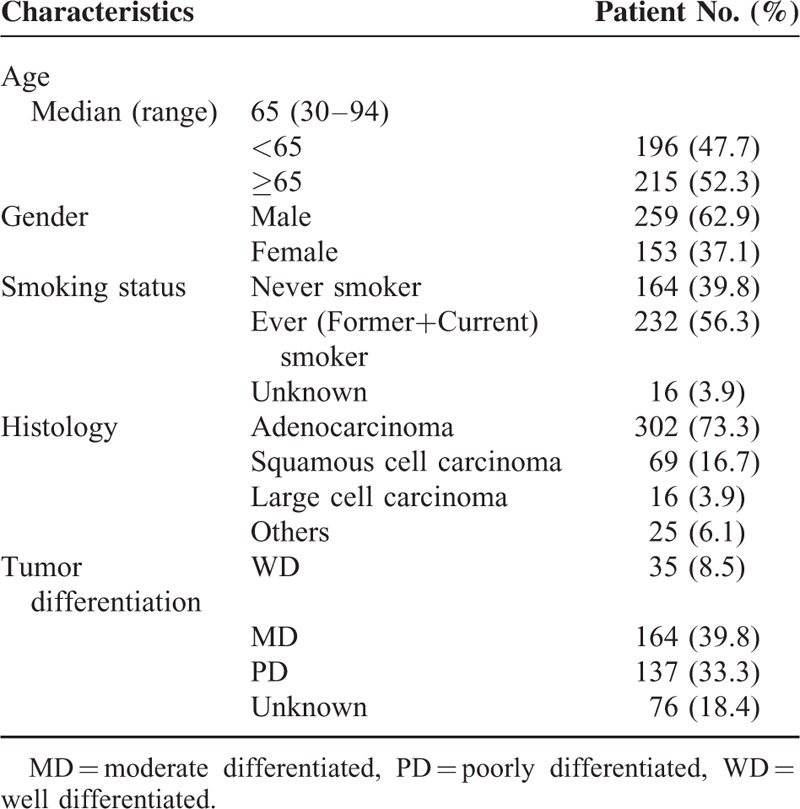
Patient and Pathological Tumor Characteristics (N = 412)

### Metabolic and Metastatic Characteristics

The distribution of SUVmax and metastatic characteristics is shown in Table 2. There was a nonnormal distribution of SUVmax, and the median value was 8.9 (range, 1–40.4). The median whole-body metastatic score was 2 (range, 1–6), and the group with the low metastatic score (score 1–2) was composed 57.5% of the patients. Sixty-six (16%) patients had cN0 LN status. Among the whole-body metastatic sites, pulmonary (lung to lung/lymphangitic spread) and skeletal metastases were the most common (58.7% and 56.1%, respectively).

**TABLE 2:**
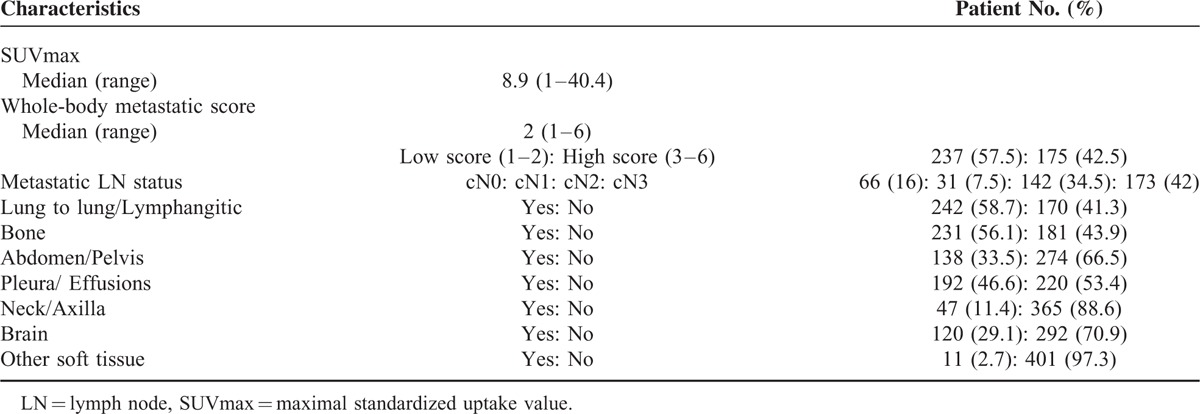
Metabolic and Metastatic Characteristics (N = 412)

### Correlation Between SUVmax and Tumor Characteristics

We explored the clinical correlation between SUVmax and tumor characteristics. The results are shown in Table 3. In nonparametric analyses, male sex (*P* = 0.001), ever smoker (*P* = 0.001), squamous cell carcinoma (*P* *<* 0.001), and poorly differentiated tumor (*P* = 0.01) had significantly higher SUVmax.

**TABLE 3:**
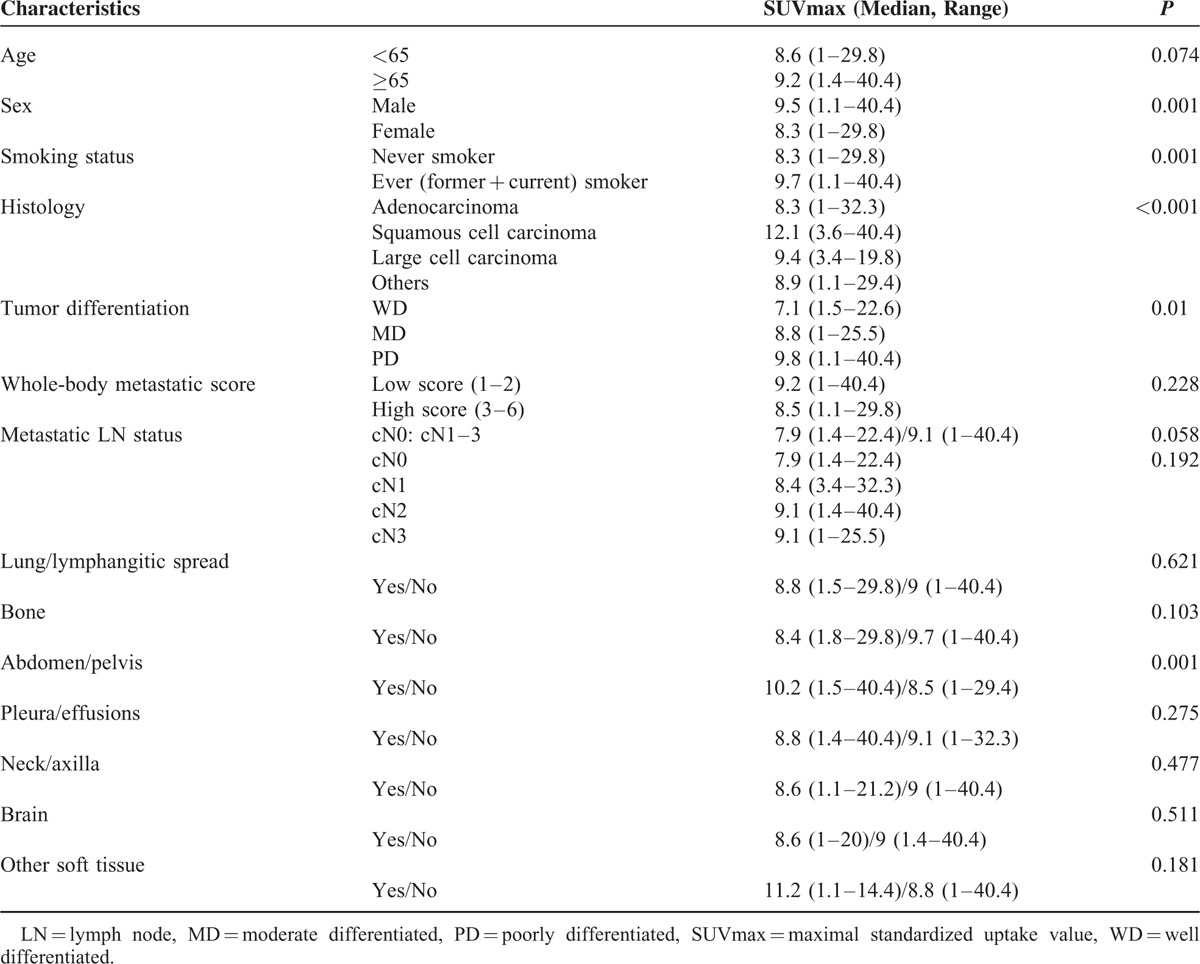
Clinical Correlation Between SUVmax and Tumor Characteristics (N = 412)

### Correlation Between SUVmax and Metastatic Region

The results of the clinical correlation between SUVmax and metastatic extent are shown in Table 3. In terms of metastatic distribution, only the abdomen/pelvis metastasis (*P* = 0.001) presented with a significantly higher SUVmax, and the positive LN group (*P* = 0.058) showed a trend toward higher SUVmax. The whole-body metastatic score and other metastatic sites showed no significant difference. Among the abdomen/pelvis metastases, liver metastasis had a significantly higher SUVmax (*P* = 0.027).

### Subgroup Analysis: Nonsquamous NSCLC

In our analysis (Table 3), we found that squamous cell carcinoma had a significantly higher SUVmax. Therefore, we hypothesized that tumors with nonsquamous NSCLC might have differential characteristics with respect to the SUVmax. SUVmax was classified into groups of the top 30 percentiles and the low 70 percentiles. The cutoff of SUVmax for the top 30 percentile was 11.4. In a subgroup of nonsquamous NSCLC (Table 4, n = 343), subpopulations with the top 30 percentiles of SUVmax were more frequently found in patients with positive metastatic LN status (*P* = 0.03) and abdomen/pelvis metastasis (*P* = 0.001). In contrast, patients with bone metastases had a significantly lower proportion of top 30 percentiles of SUVmax (*P* = 0.027).

**TABLE 4:**
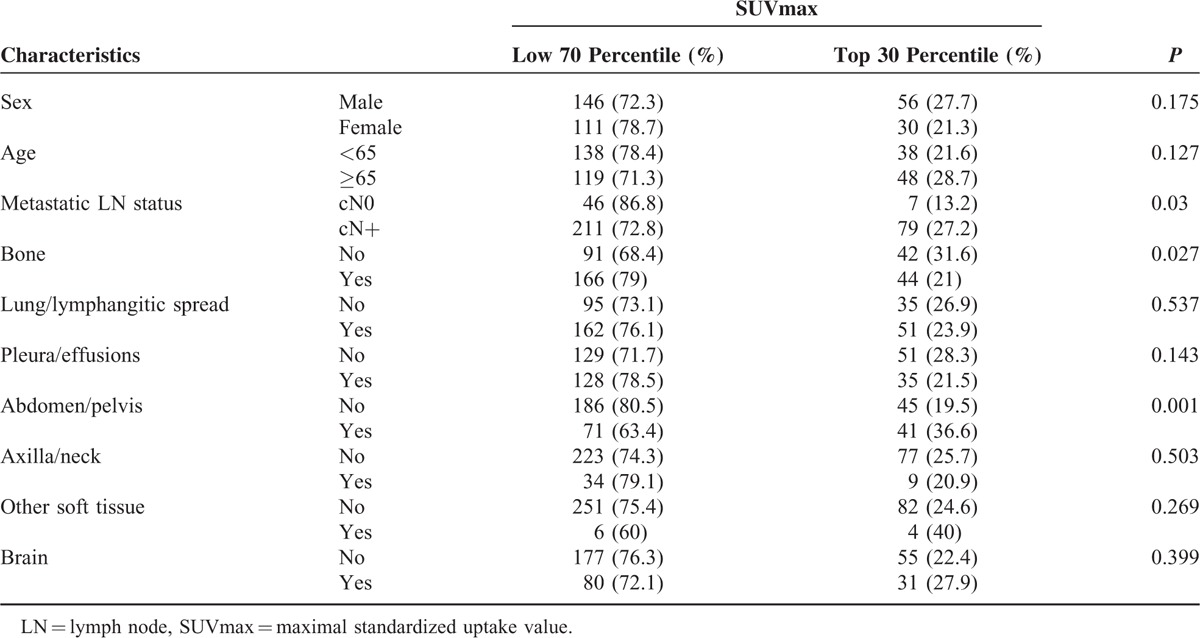
Comparison of Metastatic Tumor Characteristics According to the SUVmax in Subgroups of Nonsquamous NSCLC (n = 343, Top 30% of SUVmax vs Low 70% of SUVmax)

### Identifying Clinical Factors Associated With High SUVmax Group

To identify independent factors associated with high SUVmax, we conducted univariate and multivariate logistic regression analyses in the entire population (results not shown in the table) and in the subgroup of nonsquamous NSCLC (Table 5).

**TABLE 5:**
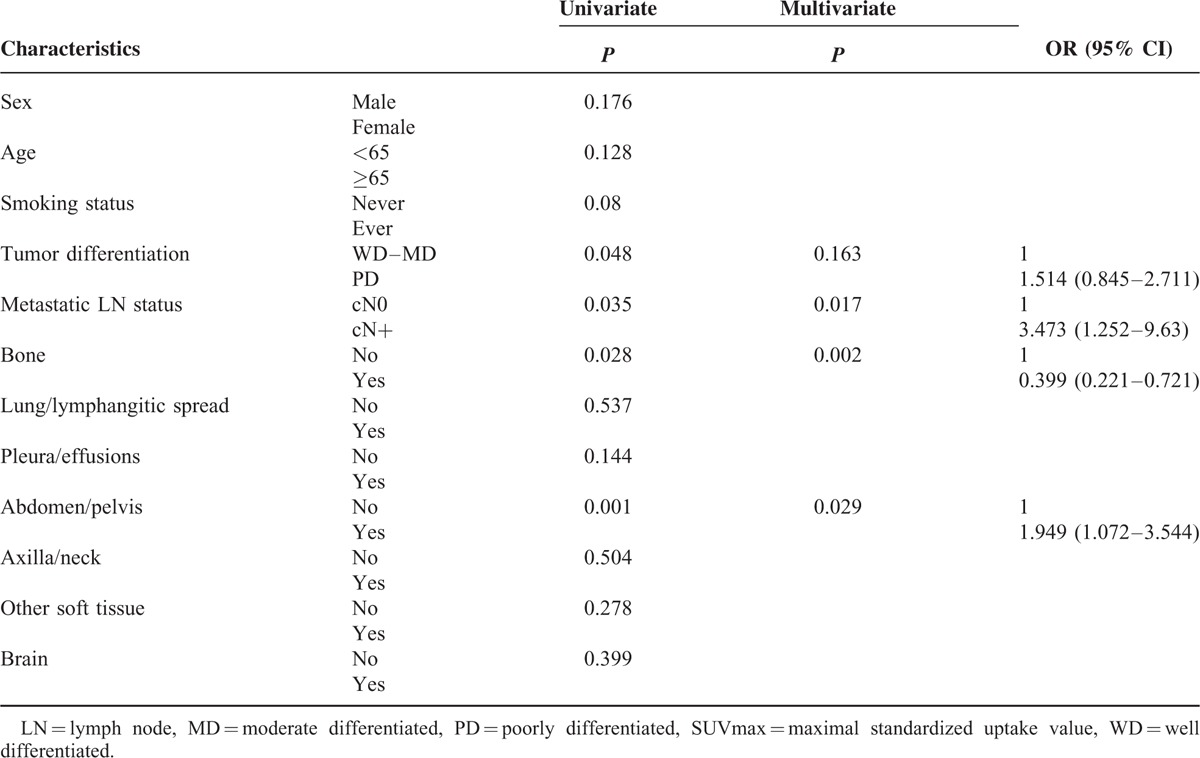
Logistic Regression Analysis for Identifying Significant Factors Associated With High SUVmax (≥11.4, Top Rank 30%) in Subgroups of Nonsquamous NSCLC

In the entire population of 412 patients, squamous cell histology (*P* < 0.001), the absence of bone metastasis (*P* = 0.006), and the presence of abdomen/pelvis metastasis (*P* = 0.008) were significantly associated with the high SUVmax group (top 30 percentile) in multivariate analysis.

We performed subgroup analysis of nonsquamous NSCLC patients (n = 343, Table 5). In multivariate analysis, positive metastatic LN status (*P* = 0.017), the presence of abdomen/pelvis metastasis (*P* = 0.029), and the absence of skeletal metastasis (*P* = 0.002) remained significant elements that predict high SUVmax. Among 3 metastatic distribution sites (LN, bone, abdomen/pelvis), bone metastasis was the most significantly correlated factor. The odds ratio for positive bone metastasis was 0.399 (95% confidence interval [CI], 0.221–0.721) in relation to the absence of bone metastasis.

The distribution of metastases among patients with nonsquamous NSCLC was divided into the following 4 groups: both abdomen/pelvis and bone metastasis (n = 73, 21.3%); abdomen/pelvis metastasis but not bone metastasis (n = 39, 11.4%); bone metastasis but not abdomen/pelvis metastasis (n = 136, 39.7%); and neither abdomen/pelvis nor bone metastasis (n = 95, 27.7%). The median SUVmax for each group was 8.5 (range, 4.2–29.8), 11.3 (range, 1.5–32.3), 8.3 (range, 1.8–29.4), and 7.8 (range, 1–22.6), respectively (*P* = 0.002, Kruskal–Wallis test).

### Prognostic Value for Survival Estimation

**FIGURE 1:**
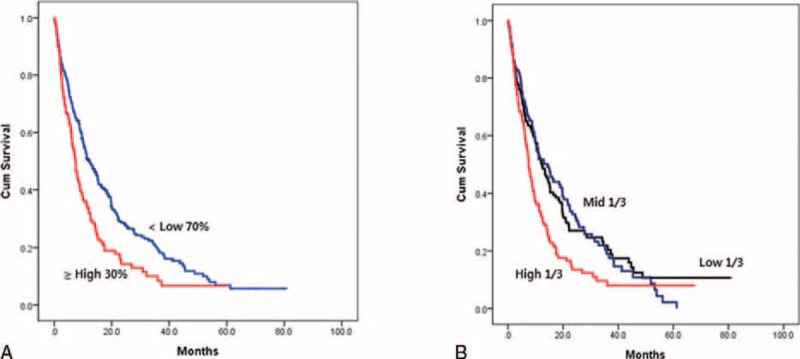
Kaplan–Meier overall survival curves. (A) Overall survival of the top 30 (≥11.4) versus the low 70 percentiles (<11.4) of SUVmax (log-rank test, *P* < 0.001). (B) Overall survival of the top 1/3 (≥10.7), mid 1/3 (≥7.2 and < 10.7), and low 1/3 of SUVmax (<7.2) (log-rank test, *P* = 0.001). SUVmax = maximal standardized uptake value.

To evaluate whether SUVmax could be an indicator of OS, we performed survival analysis between the groups in the top 30 percentiles of SUVmax and the low 70 percentiles of SUVmax. In Kaplan–Meier analysis, OS in the top 30 percentiles of SUVmax was significantly lower than in the low 70 percentiles of SUVmax (*P* < 0.001, HR 1.543, 95% CI 1.225–1.943; Figure 1). The median estimated OS time was 7.4 and 12.1 months, respectively. When we performed survival analysis of the 3 subgroups of SUVmax (cutoff values of 7.2 and 10.7), the curves again showed a significant difference (*P* = 0.001). However, there was no significant survival difference between the groups in the mid one-third of SUVmax and the low one-third of SUVmax.

## DISCUSSION

We designed this study to determine the clinical value of SUVmax in metabolic imaging of advanced stage IV NSCLC. The study population was composed of 412 stage IV NSCLC patients with available pretreatment information on 18(F)-FDG-PET/CT SUVmax. The purpose of the study was to evaluate whether tumors with high SUVmax present distinctive characteristics with respect to the metastatic distribution, tumor pathology, or OS. Our prior study has shown that metastatic extent predicts survival outcome in stage IV nonsquamous cell NSCLC.^15^ Despite the several limitations in the scoring system, the categorization of metastatic extent correlated well with survival. A previous study also showed a close correlation between tumor marker values and whole-body metastatic score.^16^ In addition, we examined the metastatic LN status to evaluate the correlation between SUVmax and LN status in this study. The SUVmax was significantly different based on the sex, smoking status, histological subtype, tumor differentiation, and several metastatic sites. However, there was no close relationship to the whole-body metastatic score. Multivariate analysis showed that high SUVmax (top 30 percentiles, ≥11.4) was independently associated with squamous cell histology, the absence of skeletal metastasis, and the presence of abdomen/pelvis metastasis in the entire group. We hypothesized that there might exist unique characteristics in the high SUVmax group in accordance with the different histological types and performed a subgroup analysis of nonsquamous NSCLC. The presence of abdomen/pelvis metastasis and the absence of skeletal metastasis again demonstrated an independent association with high SUVmax. In addition, metastatic LN status was also positively correlated with high SUVmax. In terms of survival outcome, the group with high SUVmax had a significantly poorer OS duration. Based on the study results, we identified a strong predictive value of high SUVmax for specific metastatic spread and a prognostic value for OS.

We initially hypothesized that tumors with high SUVmax would have aggressive oncologic features because many prior studies have shown poor prognosis in tumors with high SUVmax. Although the whole-body metastatic score had no correlation with SUVmax, unfavorable metastatic sites such as abdomen/pelvis metastasis or positive LN status in lung cancer were found to have an independent association with high SUVmax in the nonsquamous NSCLC subgroup. Importantly, a strong association between the absence of bone metastasis and high SUVmax was detected.

With respect to the OS time, our study supports a strong prognostic value of SUVmax in stage IV NSCLC. Although a significant association between squamous histology and high SUVmax was observed, nonsquamous cell tumors with high SUVmax also had several adverse tumor characteristics. Therefore, tumors with high SUVmax may have unique and independent unfavorable tumor features irrespective of the histological subtypes.

The cutoff values of SUVmax have been different among other prior studies. Some studies^9,17^ (SUVmax 10 and 9) found that the median SUVmax was prognostic but not in another study^11^ (SUVmax 11.1). Other studies^18–20^ used the best cutoff values (SUVmax 15 and 11) or the value conventionally used in previous studies (SUVmax 10), and revealed prognostic significance. However, the enrolled advanced stages (mixed III or IV) and treatment factors were different. Although the median SUVmax (8.9) also separated the OS curves (log-rank test, *P* = 0.018), the cutoff value of 11.4 (top 30 percentile) performed better to predict the separation of curves (log-rank test, *P* < 0.001) in our study.

Nguyen et al^21^ reported that FDG uptake is more valuable than glucose transporter type 1 (GLUT-1) or Ki-67 expression in terms of predicting prognosis in resected NSCLC patients. They concluded that FDG uptake is the most valuable prognostic indicator of tumor recurrence. Primary tumor FDG uptake was an independent predictive factor for regional LN metastasis in patients with NSCLC in a study by Li et al,^22^ who examined 80 NSCLC patients undergoing surgical LN staging procedures. In this study, there was a significant correlation between the SUVmax of the primary tumor and the pathologic N stage, and the results were concordant with ours. Chiu et al^2^ examined 152 lung adenocarcinoma patients, and found a significant difference in SUVmax among the different histological subtypes, even within the adenocarcinoma. Tumors that were solid and predominantly adenocarcinoma and non-terminal respiratory unit (TRU) type adenocarcinoma had significantly higher SUVmax, and GLUT-1 expression was statistically higher in non-TRU type tumors with a solid growth pattern.

There have been no clinical studies that explored the relationship between distinctive metastatic characteristics and SUVmax. Many previous trials only focused on the prognostic or predictive value of SUVmax, not on unique features at the time of diagnosis. The results in the present study that indicates poor OS outcome with high SUVmax are concordant with previous reports.^7,23^ The main strengths of this study are the homogeneous cohorts with stage IV NSCLC and the precise analysis for metastatic characterization. Although specific therapeutic details and an independent value predictive of OS could not be determined because of the retrospective nature of the study, we demonstrated a strong association between high SUVmax and poor outcome based on statistical analysis.

A limitation of the categorization of whole-body metastatic score is that the scoring system was based only on metastatic regions, not the exact entire tumor burden in each subregion. The metastatic LN status and other metastatic sites were assessed according to the clinical findings of metabolic imaging studies. When accurate discrimination of primary tumors was challenging, we used the highest value of SUVmax measured from tumors in the thorax. In addition, the shorter survival rate in the high SUVmax group could be affected by the clinical decision of medical oncologist rather than the unique prognostic power of SUVmax (because of the selection bias of treatment factors caused by SUVmax).

In conclusion, tumors with high SUVmax in advanced NSCLC had a strong correlation with squamous cell histology and adverse metastatic characteristics in a subgroup of nonsquamous NSCLC. The study results that stage IV nonsquamous cell tumors with high SUVmax were independently associated with abdomen/pelvis metastasis, positive LN status, and the absence of bone metastasis appear to be novel findings. We also observed that tumors with high SUVmax had a significantly detrimental effect on OS. The tumors with different SUVmax may have distinctive metastatic and biological features, and further studies are necessary to demonstrate the underlying mechanisms of biology in metabolic imaging.
